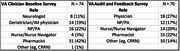# Examining Barriers and Strategies to Improve Alzheimer's Disease Care in the Veterans Affairs Setting

**DOI:** 10.1002/alz70858_102944

**Published:** 2025-12-24

**Authors:** Arushi Kapoor, Jeffrey Carter, Emma Landau, Chris Napolitan, Laura Simone, Larissa Jarzylo, Andrew E. Budson

**Affiliations:** ^1^ Penn Memory Center, Philadelphia, PA, USA; ^2^ PRIME Education, New York, NY, USA; ^3^ Boston University Alzheimer's Disease Research Center, Boston, MA, USA

## Abstract

**Background:**

The recent introduction of disease modifying therapies (DMTs) for Alzheimer's disease (AD) offers patients new hope to slow disease progression. Early diagnosis is crucial because DMTs show greater efficacy in individuals with earlier stages of AD. Veterans have a higher prevalence of risk factors for the development of AD and other dementias. This study examined barriers to and facilitators of optimal AD evaluation and management in Veterans Affairs (VA) heathcare settings.

**Methods:**

This qualitative study administered surveys to clinicians in 5 VA‐affiliated clinics between March 2024 and October 2024 as part of a quality improvement program. Clinical teams at each site participated in an audit‐feedback session to review baseline data and develop action plans. A follow‐up survey was administered after 3 months to assess process improvements.

**Results:**

A total of 144 surveys were collected from VA clinicians at baseline and during audit‐feedback sessions (Table). Difficulty diagnosing AD and time constraints related to discussion of treatment options were the greatest challenges in managing AD among identified by providers. Clinicians identified patient hesitancy in discussing symptoms of dementia and challenges in distinguishing between normal aging from dementia symptoms as top factors which contributed to delayed diagnosis of AD. In patients diagnosed with early AD, difficulties with care coordination and patients’ mental (eg cognitive impairment) or social issues (eg behavioral changes) were the main reasons eligible patients did not receive DMTs. Clinical pathway or formulary restrictions and lack of time to align treatment paradigms with emerging evidence were identified as additional challenges to administering DMTs to eligible patients. Following the sessions, process changes included implementation of a cognitive testing workflow, improvements to documentation of screening and assessment in the EMR, education for clinicians to support shared treatment decision‐making with patients, and formation of a dementia care working group.

**Conclusions:**

These findings highlight barriers in early diagnosis and management of AD in the VA setting. Action plans designed to address these barriers have resulted in implementation of process improvements.